# The human alpha7 nicotinic acetylcholine receptor is a host target for the rabies virus glycoprotein

**DOI:** 10.3389/fcimb.2024.1394713

**Published:** 2024-05-21

**Authors:** Brittany C. V. O’Brien, Shelly Thao, Lahra Weber, Helen L. Danielson, Agatha D. Boldt, Karsten Hueffer, Maegan M. Weltzin

**Affiliations:** ^1^ Department of Chemistry and Biochemistry, University of Alaska Fairbanks, Fairbanks, AK, United States; ^2^ Department of Veterinary Medicine, University of Alaska Fairbanks, Fairbanks, AK, United States

**Keywords:** nicotinic acetylcholine receptor, nAChR, rabies virus, alpha7, RVG, electrophysiology, N2a, rabies virus glycoprotein

## Abstract

The rabies virus enters the nervous system by interacting with several molecular targets on host cells to modify behavior and trigger receptor-mediated endocytosis of the virion by poorly understood mechanisms. The rabies virus glycoprotein (RVG) interacts with the muscle acetylcholine receptor and the neuronal α4β2 subtype of the nicotinic acetylcholine receptor (nAChR) family by the putative neurotoxin-like motif. Given that the neurotoxin-like motif is highly homologous to the α7 nAChR subtype selective snake toxin α-bungarotoxin (αBTX), other nAChR subtypes are likely involved. The purpose of this study is to determine the activity of the RVG neurotoxin-like motif on nAChR subtypes that are expressed in brain regions involved in rabid animal behavior. nAChRs were expressed in *Xenopus laevis* oocytes, and two-electrode voltage clamp electrophysiology was used to collect concentration-response data to measure the functional effects. The RVG peptide preferentially and completely inhibits α7 nAChR ACh-induced currents by a competitive antagonist mechanism. Tested heteromeric nAChRs are also inhibited, but to a lesser extent than the α7 subtype. Residues of the RVG peptide with high sequence homology to αBTX and other neurotoxins were substituted with alanine. Altered RVG neurotoxin-like peptides showed that residues phenylalanine 192, arginine 196, and arginine 199 are important determinants of RVG peptide apparent potency on α7 nAChRs, while serine 195 is not. The evaluation of the rabies ectodomain reaffirmed the observations made with the RVG peptide, illustrating a significant inhibitory impact on α7 nAChR with potency in the nanomolar range. In a mammalian cell culture model of neurons, we confirm that the RVG peptide binds preferentially to cells expressing the α7 nAChR. Defining the activity of the RVG peptide on nAChRs expands our understanding of basic mechanisms in host-pathogen interactions that result in neurological disorders.

## 1 Introduction

The molecular interactions between pathogens and host targets can result in behavioral modifications and neurological disorders by poorly understood mechanisms (Moore, 2013; Webster et al., 2013; Elbirt et al., 2015; Jackson, 2016a). The rabies virus, a member of the family *Rhabdoviridae, genus Lyssavirus*, is one pathogen that affects animal behavior, and is of health relevance as it infects and kills 50,000 to 70,000 humans globally each year (Hampson et al., 2015). Rabies infection has a case fatality rate approaching 100% due to a lack of satisfactory treatment options once the virus has entered the central nervous system (CNS) and symptoms appear (Jackson, 2009). The rabies virus infects host cells via specific receptor interactions facilitated by the rabies viral glycoprotein (RVG) (Nisole and Saib, 2004; Lindemann et al., 2013; Hansen et al., 2017). RVG interacts with host receptors, including nicotinic acetylcholine receptors (nAChRs) (Lentz et al., 1983), neural cell adhesion molecule (Thoulouze et al., 1998), p75 neurotrophin receptor (Tuffereau et al., 1998), metabotropic glutamate receptor subtype 2 (Wang et al., 2018), and integrin β1 (Shuai et al., 2020) to initiate viral endocytosis into the cell (Lafon, 2005; Fooks et al., 2017).

The neuromuscular nAChR is a cell-surface receptor target for the rabies virus by direct interactions with the RVG (Lentz et al., 1983). The nAChR receptor family is a diverse set of pentameric complexes composed of different combinations of subunits (α1 - α10, β1 - β4, δ, γ, and ε), and responds to the neurotransmitter acetylcholine (ACh) (Gotti et al., 2009; Wu and Lukas, 2011). The subunit composition determines the pharmacological and biophysical properties of these receptors, and individual receptor subtypes are associated with a wide variety of animal behavior and biological processes (Gotti et al., 2009; Wu and Lukas, 2011; Hurst et al., 2013).

Specific neuronal nAChR subtypes modulate behavioral and physiological processes that are also altered during rabies infection (Champtiaux et al., 2003; Hogg et al., 2003; Grady et al., 2007; Gotti et al., 2010). Rabies-associated behaviors include aggression, anxiety, hyperactivity, loss of motor control, and changes in cardio-respiratory control (Rupprecht et al., 2002; Jackson, 2016b; WHO, 2017). α7 nAChRs are one of the most abundantly expressed brain subtypes and are enriched in the cortical and limbic regions, especially the prefrontal cortex, hippocampus, and amygdala areas of the mammalian brain (Albuquerque et al., 1995; Fabian-Fine et al., 2001a; Gotti and Clementi, 2004; Dickinson et al., 2008; Lester, 2014). The loss of α7 nAChR subtype function, either by pharmacological means in mice or by a genetic deletion in humans, as occurs with 15q13.3 microdeletion syndrome, increases aggression (Shinawi et al., 2009; Fejgin et al., 2014; Lewis et al., 2018).

The brainstem expresses α3β2 and α3β4 nAChR subtypes and has integrative functions including controlling the cardiovascular system, respiratory control, awareness, consciousness, and muscle control (Champtiaux et al., 2003; Hogg et al., 2003; Gotti et al., 2005). Locomotor behavior involves the nigrostriatal and mesolimbic dopaminergic systems, brain systems that express the α4α6β2β4, α6β2β3, and α4β2α5 nAChR subtypes (Luetje, 2004; Salminen et al., 2004; Grady et al., 2007; Gotti et al., 2010; Quik et al., 2011). It remains unknown if these nAChRs have modified function in the presence of RVG. In contrast, the α4β2 nAChR subtype, the most common nAChR in the CNS (Wada et al., 1989; Zoli et al., 1998), is a functional target for RVG peptides (Hueffer et al., 2017).

The RVG contains a “neurotoxin-like” motif that is prevalent in small three-fingered proteins, including snake α-neurotoxins, CNS prototoxins, and α-conotoxins (Galat et al., 2008; Azam and McIntosh, 2009). The three-fingered protein domain fold contains a pattern of three to four disulfide bonds, which results in defined structural loops (“fingers”) that are rich in beta-sheets and are critical for interactions with defined nAChR subtypes. The RVG neurotoxin-like motif is located between residues 175 - 203 and has high sequence homology to loop 2 (the central loop) of the snake α-neurotoxins, α-conotoxins, and prototoxins (**Table 1**). This region is at the surface of the glycoprotein and is favorably positioned to interact with nAChRs (Yang et al., 2020). Structural studies of the snake neurotoxin αBTX, which is an α7 subtype-selective antagonist, indicate that residues homologous to the RVG neurotoxin-like motif interact with the α7 nAChR subtype in the orthosteric binding site (Antil-Delbeke et al., 2000; Noviello et al., 2021). Eight of these 11 residues found within the RVG neurotoxin-like motif (C189 - R199) are conserved between RVG and at least one snake toxin that targets nAChRs (**Table 1**).

**Table 1:** Sequence alignment of the neurotoxin-like motif of the RVG and three nAChR targeting snake neurotoxins.

Virus/Toxin	Sequence																														
RVG	**Y**	T	–	I	**W**	M	P	E	N	P	R	L	G	T	S	**C**	**D**	I	**F**	T	N	**S**	**R**	**G**	**K**	**R**	A	S	K	**G**	203
α-Bungarotoxin	**Y**	R	K	M	**W**	–	–	–	–	–	–	–	–	–	–	**C**	**D**	A	**F**	C	S	**S**	**R**	**G**	**K**	V	V	E	L	**G**	43
α-Cobratoxin	**Y**	T	K	T	**W**	–	–	–	–	–	–	–	–	–	–	**C**	**D**	A	**F**	C	S	I	**R**	**G**	**K**	**R**	V	D	L	**G**	40
κ-Bungarotoxin	F	L	K	A	Q	–	–	–	–	–	–	–	–	–	–	**C**	**D**	K	**F**	C	S	I	**R**	**G**	P	V	I	E	Q	**G**	40

Residues homologous between the RVG (GenBank ID: APD77164.1) and at least one snake neurotoxin (loop 2) are bolded and highlighted in grey. Neurotoxin loop 2 regions are involved in binding to nAChRs.

Specific nAChR subtypes may be important during rabies virus infection, and individual residues in the neurotoxin-like motif of RVG are likely mediators of functional activity. We hypothesize that the RVG peptide inhibits the activity of nAChRs, with a preference for the α7 subtype, due to the sequence homology of the RVG neurotoxin-like motif and αBTX. Furthermore, RVG residues phenylalanine (F) 192, serine (S) 195, arginine (R) 196, and R199 may be important in influencing peptide potency, as these residues are highly conserved among neurotoxins and are central for αBTX binding (Noviello et al., 2021). Using two-electrode voltage clamp (TEVC) electrophysiology, we define nAChR selectivity and mechanisms of activity of the RVG peptide and identify specific peptide residues that are important for determining apparent potency. We further provide evidence that the RVG peptide mirrors the actions of the rabies ectodomain. Using a cell culture model of neurons, we visualize the RVG peptide preferentially interacting with cells expressing the α7 subtype of nAChRs. Our presented work improves our understanding of the cellular mechanisms altered by rabies infection and has identified a potential new therapeutic target.

## 2 Materials and methods

### 2.1 Reagents

ACh (Cat# A6625), atropine (Cat# A0257), and bovine serum albumin (BSA) (Cat# A7030) were purchased from Sigma-Aldrich (St. Louis, MO). Methyllycaconitine (MLA, Cat# ab120072) was purchased from Abcam (Boston, MA). RVG peptides (>90% HPLC purity) were synthesized by ELIM Biopharmaceuticals, Inc. (Hayward, CA). Lyophilized peptides were stored at -4°C until use. For use on cultured mammalian cells, peptides were initially dissolved in dimethyl sulfoxide (DMSO) (Sigma-Aldrich), before dilution with cell media. The rabies virus glycoprotein ectodomain (RVGE, Pasteur variant, Met1 - Leu524) (>85% SDS-PAGE purity) (Cat# PX-P6266) was purchased from ProteoGenix (Miami, FL). All other reagents used to make oocyte incubation and electrophysiology buffers were from Sigma-Aldrich unless otherwise specified. Fresh solution stocks were made daily and diluted as required.

To culture Neuro-2a (N2a) cells (ATCC Cat# CCL-131, RRID: CVCL_0470) purchased from American Tissue Culture Collection (ATCC, Manassas, VA), Eagle’s Minimum Essential Medium (EMEM, ATCC, Cat# 30–2003), fetal bovine serum (FBS, Cat# 97068–085), Penicillin-Streptomycin (Cat# K952–100ML), and pH 7.4 phosphate-buffered saline (PBS, Cat# 114–056-101) were purchased from VWR (Radnor, PA). Gibco Opti-Minimal Essential Medium (Opti-MEM, Cat# 31985–070) was purchased from Gibco ThermoFisher Scientific (Waltham, MA) and Trypsin-EDTA (0.25%) from ATCC (Cat# 30–2101). Lipofectamine 2000 Transfection Reagent was purchased from Invitrogen (ThermoFisher Scientific, 11668–019).

### 2.2 DNA constructs and cRNA synthesis

The human α3, α4, α6/α3 chimera, β2, β3, and β4 nAChR subunits were previously cloned into the pCI mammalian expression vector (Cat# E1731 Promega, Madison, WI) (George et al., 2012; Eaton et al., 2014; Luo et al., 2014). The α7 subunit and the concatenated β3-α6-β2-α4-β2 nAChR were subcloned into pSHE and pSGEM AMV mammalian expression vectors (modified pGEMHE vectors), respectively. All the above-listed cDNAs were a generous gift from Drs. Ronald J. Lukas (Barrow Neurological Institute, St. Joseph’s Hospital, Phoenix, AZ) and Paul Whiteaker (Virginia Commonwealth University, Richmond, VA). The α5 subunit DNA was synthesized and cloned into the pMA *E. coli* expression vector (ThermoFisher Scientific, Waltham, MA). Using the XhoI (Cat# R0146L) and NotI-HF (Cat# R3189L) enzyme restriction sites, the α5 subunit was subcloned into the pCI vector. The α6/α3 subunit chimera, composed of the extracellular domain of α6 followed by the transmembrane and intracellular domains of the α3 subunit, has increased expression compared to native α6 subunits while retaining α6-like pharmacology (Kuryatov et al., 2000). To ensure the exact stochiometric expression of the β3-α6-β2-α4-β2 nAChR, subunits are linked by six to 12 alanine-glycine-serine (AGS) repeats (Kuryatov and Lindstrom, 2011).

Upon arrival, lyophilized DNA was reconstituted in nuclease-free water, and single subunit constructs were transformed into 5-α (Cat# C2987H), or in the case of the larger β3-α6-β2-α4-β2 construct, 10-β competent *E. coli* cells (Cat# C3019I, New England Biolabs, Ipswich, MA) for large-scale production of cDNA. Each cDNA was isolated using QIAprep Spin Miniprep kits (Cat # 27106; Qiagen, Valencia, CA), and verified using restriction enzymes and gel electrophoresis. To prepare for cRNA synthesis, cDNAs were linearized using restriction enzymes (New England Biolabs) SwaI (Cat# R0604S) or NheI-HF (Cat# R3131S). Following cDNA linearization, samples were treated with proteinase K (30min at 50°C) (Cat# P8107S, New England Biolabs) and purified using Qiagen’s PCR clean-up kit (Cat# A9282). cRNAs were transcribed using the T7 mMESSAGE mMACHINE™ High Yield Capped RNA Transcription Kit (Cat# AM1344, ThermoFisher Scientific). The cRNA purity was confirmed by measuring the 260/280 ratio via a Nanodrop 2000 (ThermoFisher Scientific, Waltham, MA) and by visual inspection of samples run on a 1% agarose gel. The samples were sub-aliquoted and stored at -80°C.

To express human α7 nAChRs in N2a cells, the α7 and α7-pHuji subunit plasmid DNAs were prepared and verified as previously described in O’Brien et al., 2023 (O'Brien et al., 2023). The α7 nAChR chaperone protein NACHO pREP9 DNA plasmid was generously gifted by Dr. R. Loring (Northeastern University, Boston, MA). To achieve transfection-grade, endotoxin-free DNA for use in mammalian cell culture experiments, α7, α7-pHuji, and NACHO plasmid DNAs were extracted from 10-β competent *E. coli* cells (New England BioLabs Inc.) grown in Circle Grow broth media (MP Biomedicals, Santa Ana, CA, Cat# 3000132) with 0.1 mg/mL ampicillin using the EndoFree Plasmid Maxi Kit (Qiagen, Valencia, CA, Cat# 12362). cDNA concentrations and purity (260/280 ratio) were measured using the Thermo Scientific NanoDrop 2000 spectrophotometer and visually verified using 1% agarose gel electrophoresis. cDNAs were aliquoted and were kept frozen at -4°C until use.

### 2.3 Oocyte preparation and cRNA injection

*Xenopus laevis* oocytes were harvested either in-house (IACUC Assurance ID D16–00482) or purchased from EcoCyte Bioscience (IACUC certified, Austin, TX). Briefly, in-house oocyte extractions were initiated with each animal being anesthetized by exposure to a sterile bath of 2 g/L of Tricaine methanesulfonate (MS-222) (Cat # E10521, Sigma-Aldrich) dissolved in sterile “frog water” (156 g of Instant Ocean Sea Salt (Cat# SS15–10, Instant Ocean, Blacksburg, VA) dissolved into 120 L reverse osmosis water, pH using sodium bicarbonate to 7.0 - 7.5) until the animal lost its righting reflex and had no response to toe pinches (approximately 15min). Using sterile procedures, oocytes were surgically extracted and placed in sterile Ca^2+^-free oocyte incubation buffer (82.5 mM NaCl, 2.5 mM KCl, 1 mM MgCl_2_•6H_2_O, 1 mM Na_2_HPO_4_, 5 mM HEPES, pH to 7.5 using NaOH). The surgical site was cleaned, sutured closed, and the animal was placed in a sterile recovery tank until she could breathe and move independently. The animal was then placed in a separate housing chamber for two days, receiving daily sterile frog water changes and food, before returning to the recirculating water housing facility. The animal remained in isolation for two weeks to monitor the surgical site before rejoining the frog colony. Animals received a total of four surgeries, two on either side, before being euthanized on the fourth surgery by an overdose of MS-222 and removal of the heart. All animals were housed on a 12/12 light/dark cycle, in a 21 - 22°C room, with daily UV treatment and filtration of the water, water quality monitoring, and feeding.

Isolated oocytes were defolliculated using 2 mg/ml of collagenase A (Cat# 11088793001, Roche, Indianapolis, IN) in a Ca^2+^-free incubation buffer for approximately 1hr at room temperature using a rocker. Oocytes were washed with sterile incubation buffer (82.5 mM NaCl, 2.5 mM KCl, 1 mM MgCl_2_•2H_2_O, 1 mM Na_2_HPO_4_, 5 mM HEPES, 600 μM theophylline, 2.5 mM Na pyruvate, 50 U/ml penicillin, 50 µg/ml streptomycin, 50 µg/ml neomycin, 50 µg/ml gentamycin sulfate, and pH to 7.5 using NaOH) and stored in a 13°C incubator until ready for cRNA injections.

Using only stage V oocytes, nAChR subtypes, and isoforms were expressed in *Xenopus laevis* oocytes using cRNA microinjections. Isoforms that contain two α subunits and three β subunits were achieved using a 1: 30 α: β cRNA injection ratio, or in a 30: 1 α: β cRNA injection ratio for isoforms that contain three α and two β subunits (Nelson et al., 2003; Weltzin et al., 2016). The exception to this is the (α4β2)_2_α4 subtype which was expressed using a 100: 1 α4: β2 cRNA injection ratio. Expression of the α6/α3β2β3 and α4β2α5 isoform was achieved using a ratio of 2: 2: 1 α6/α3: β2: β3 or 1: 1: 10 α4: β2: α5 cRNA, respectively (Broadbent et al., 2006; Dash et al., 2012; Dash et al., 2014). The total ng of injected material is presented in **Supplementary Table 1**. A total of 40 ng of α7 and 30 ng of β3-α6-β2-α4-β2 cRNA resulted in robust expression of these nAChR subtypes. In all cases, 81 nL of cRNA were injected into each oocyte by a pulled micropipette with an outer diameter of about 40 nm. Injected oocytes were incubated for 36hrs - 6days, before data collection.

### 2.4 Two-electrode voltage clamp electrophysiology

Using TEVC and nAChR-expressing *Xenopus laevis* oocytes, we evaluated ACh, RVG peptides (see **Table 1** for sequence), and the RVGE by voltage clamping each oocyte at -70 mV with an Axoclamp 900A amplifier (Molecular Devices, LLC, Sunnyvale, CA). Data acquisition and analysis were performed using pClamp 10.7 software (RRID: SCR_011323, Molecular Devices, LLC). Recordings were sampled at 10 kHz (low-pass Bessel filter: 40 Hz, high-pass filter: DC). Recording electrodes were pulled from thin wall capillary glass (Cat # TW100F-3, World Precision Inst, Sarasota, FL) and filled with 3 M KCl. Final electrode resistance ranged from 0.5 - 10 MΩ. Oocytes with leak currents >100 nA were discarded. The recording apparatus used minimized post-valve tubing length and a custom manifold to reduce dead volume.

Drug solutions were applied to voltage-clamped oocytes using a 16-channel, gravity-fed perfusion system with automated valve control (AutoMate Scientific, Inc., Berkeley, CA) at a rate of approximately 7 mL/min. All drug solutions were made fresh daily in an Oocyte Ringer 2 (OR_2_) recording buffer (92.5 mM NaCl, 2.5 mM KCl, 1 mM MgCl_2_•6H_2_O, 1 mM CaCl_2_•2H_2_O, 5 mM HEPES, pH to 7.5 using NaOH) containing atropine sulfate (1.5 μM) and 0.1% BSA. Atropine sulfate (1.5 μM) and 0.1% BSA were added to all recording solutions to block any potential muscarinic responses and to prevent the peptide or ectodomain from potentially sticking to the plastic components of the recording apparatus, respectively (Luo et al., 2014; O'Brien et al., 2023). Using 0.1% BSA caused no activation of the nAChR subtypes (data not shown). The RVGE came prepared in a pH 7.4, 0.01 M PBS buffer, which we diluted to the desired concentration using the OR_2_ recording buffer.

To verify that our cRNA injection ratios resulted in relatively pure nAChR isoform populations, ACh concentration-response curves were generated (**Supplementary Figure 1**, **Supplementary Table 1**). Half-log concentration ranges of ACh 0.003 μM - 10 mM, depending on receptor subtype, were applied to voltage-clamped oocytes. A recovery period of 1.5min was used between application of each ACh concentration. The ACh concentration that resulted in 90% of the maximal response (EC_90_) was determined for each nAChR subtype and used in further experiments described immediately below.

RVG peptide concentration-response data was collected by pre-incubating nAChR-expressing oocytes with increasing concentrations of the RVG peptide (0.01 - 300 μM for α7, or 0.01 - 1000 μM for all other subtypes) via automatically triggered 30s valve openings (henceforth referred to as “applications”). Each peptide application was followed by a 1s application of ACh at the EC_90_ concentration (α4β2α5 20 µM; concatenated β3-α6-β2-α4-β2 40 µM; (α4β2)_2_β2 100 µM; (α4β2)_2_α4, α6/α3β2β3, (α3β2)_2_β2 316 µM; (α3β4)_2_β4 400 µM; (α3β4)_2_α3 800 µM, α7 1.26 mM, (α3β2)_2_α3 3.16 mM). A 4min wash recovery period was used between each RVG peptide concentration. Concentrations greater than 1000 μM were not used to prevent potential non-specific effects. All responses were normalized to the initial ACh response prior to peptide exposure. α7 nAChRs were normalized to the second ACh control response as this subtype requires pre-exposure to an agonist before a full response is achievable.

To characterize the RVG peptide’s mechanism of antagonism, we performed co-application of the RVG peptide with ACh to generate concentration-response profiles (a different drug application procedure than those described in the preceding paragraph). This was accomplished by co-applying increasing concentrations of ACh (0.010 µM - 10 mM) with a single peptide concentration (10 µM, 30 µM, or 50 µM) for 1s with an 84s wash of OR2 recording buffer in between each drug application. Following the co-application responses, two applications of ACh (10 mM) that evoked the maximal response (I_max_) without peptide were performed. The first and second ACh-only responses were comparable in amplitude. Co-application data was normalized to the second ACh I_max_ response. This experiment was also performed with co-applying ACh and MLA (10 nM, 100 nM, or 1 µM) for comparison.

To test the RVG F192A, S195A, R196A, or R199A peptides on α7 nAChRs, concentration-response experiments (0.01 - 300 μM) were performed as described for the RVG peptide. Several control experiments were performed. To demonstrate that we can block α7 nAChRs, 10 nM of the competitive antagonist MLA was pre-applied to α7 nAChR-expressing oocytes for 30s, followed by ACh (EC_90_) stimulation. The RVG peptide was applied to un-injected oocytes for 2s to demonstrate that the peptide caused no effect on its own. To determine a negative control for the RVG peptide, we turned to the conotoxin literature. Conotoxins are well-studied short peptides (12 - 40 amino acids) that target nAChRs with subtype specificity. Using single point substitutions outside of the predicted interaction region and comparison to the unmutated conotoxin are used as a control for these peptides (Hone et al., 2019; Huang et al., 2022; Xu et al., 2023). In our presented work, residue S195 in the RVG peptide has been substituted with an A and the effects on the apparent potency have been evaluated.

Determination of RVGE effects on α7 nAChRs was accomplished by placing each oocyte in incubation buffer containing a single concentration of the ectodomain (1 - 300 nM) for 5min. Oocytes were then rapidly voltage clamped and the α7 nAChRs were stimulated with a 1s application of ACh EC_90_ (1.26 mM). Responses were normalized to a group of α7 nAChR-expressing oocytes that were only exposed to 1.26 mM ACh (RVGE naïve).

### 2.5 Expression of human α7 nAChRs in N2a cells

N2a cells were maintained in Eagle’s Minimum Essential Medium (EMEM) supplemented with 10% fetal bovine serum and 1X penicillin-streptomycin and incubated in a humidified cell culture incubator at 37°C and 5% CO_2_. Cells were subcultured onto poly-D-lysine-coated glass coverslips (MatTek, MA, Cat# P35GC-0–10-C) and transiently transfected when dishes were approximately 70% confluent with cells. To express α7 nAChRs, α7 or α7-pHuji and Novel Acetylcholine Receptor Chaperone (NACHO) coding plasmid DNAs were combined at a 4:1 ratio in Opti-MEM. The Lipofectamine 2000 transfection reagent was used for transient transfection, according to the manufacturer’s protocol. 24hrs post-transfection, cells were either imaged or treated with peptide for an additional 24h before live-cell confocal imaging. To ensure robust plasma membrane expression could be achieved by this procedure, we added a pH-sensitive fluorescent tag to α7 subunit C-terminus plasmid DNA. At pH 7.4 pHuji fluoresces when excited at 566 nm (emission 598 nm), and fluorescence is quenched at pH 5.0. Using this property, the location of expressed α7-pHuji nAChRs could be determined (**Supplementary Material**, **Supplementary Figure 2**).

### 2.6 RVG-FITC peptide labeling of N2a cells

N2a cells un-transfected and transiently transfected with α7 nAChR and NACHO plasmid DNAs were treated with 30 µM of the RVG peptide C-terminally tagged with the fluorophore fluorescein 5-isothiocyanate (FITC) for 24hr before visualization using a laser scanning confocal microscope (Olympus Fluoview FV10i Laser Scanning Confocal Microscope). A 24hr incubation period was used to ensure we obtained binding saturation. Prior to imaging, the cells were rinsed with PBS three times and PBS (pH 7.2) buffer was used during live-cell image acquisition. Imaging of the RVG-FITC peptide was performed using an excitation wavelength of 495 nm and an emission wavelength of 519 nm. For comparison, 80 nM Alexa Fluor 647-conjugated αBTX (αBTX-AF647, Cat# B35450, ThermoFisher) was added to the EMEM growth medium for 24hr before being imaged. For imaging of the Alexa Fluor 647-conjugate, the excitation wavelength was set to 653 nm, while the emission wavelength was 668 nm. Images were further analyzed by corrected total cell fluorescence (CTCF) analysis.

### 2.7 Confocal image processing

After capture, images used for visualization were deconvoluted using ImageJ (RRID: SCR_003070, National Institutes of Health, Bethesda, MD) and the plug-ins DeconvolutionLab 2 and PSF Generator (Biomedical Imaging Group and École polytechnique fédérale de Lausanne). Appropriate point spread functions (PSFs) were calculated using the PSF Generator and the microscope settings described in the above sections. Using the calculated PSFs and the DeconvolutionLab2 plug-in, images were deconvoluted using the Richardson-Lucy algorithm with 10 iterations (Laasmaa et al., 2011). To determine if RVG internalizes into N2a cells, z-stack images were also deconvoluted and processed to three-dimensional images as further described in the **Supplementary Materials** (**Supplementary Figure 3**).

### 2.8 Data analysis

ACh and RVG peptide half maximal effective concentrations (EC_50_), ninety- or forty-percent effective concentrations (EC_90_ or EC_40_, respectively), extrapolated half maximal inhibitory concentrations (IC_50_), 95% confidence intervals (CI), I_max_, and Hill slopes (n_H_) were determined from peak currents using individual oocytes expressing a defined nAChR population. n_H_ values are shown to reflect the relative extent of cooperativity among the ligand binding sites and to demonstrate that the curve fits are realistic. All experiments were conducted on at least two batches of cRNA synthesis and three oocyte isolations from unique individual frogs. For each set of experiments, the number of experimental replicates from unique individuals is indicated by N followed by the number of individual oocytes, n, throughout the manuscript.

Concentration-response profiles were fit using non-linear curve fitting and GraphPad Prism (v. 10) software (RRID: SCR_002798, La Jolla, CA) with standard built-in algorithms. ACh monophasic sigmoidal fits (unconstrained) or, in the case of (α4β2)_2_α4, constrained (n_H (1 and 2)_ = 1) biphasic equations, were used to fit all parameters. The (α4β2)_2_α4 isoform concentration-response curve constraint is justified by this isoform having two ACh potencies (Nelson et al., 2003; Gotti et al., 2008; Carbone et al., 2009; Eaton et al., 2014; Weltzin et al., 2019). A sum-of-squares F-test was used to ensure data was best fit with a biphasic model. Competitive antagonist data was fit using an unconstrained monophasic sigmoidal equation. RVG peptide and RVGE data were fit with monophasic sigmoidal inhibition curves to determine the parameters. In several cases, RVG peptides did not fully inhibit the examined nAChRs. To determine the RVG peptide and RVGE potencies, the bottom of each inhibition curve was fixed to 0 to facilitate curve fitting. Several subtypes had poor curve fits (r^2^ < 0.8), and thus the potencies could not be accurately determined.

CTCF was determined using ImageJ software. The mean integrated density of fluorescence was calculated for each experimental group using non-processed static images. To minimize observer bias, the observer remained blind to the fluorescence channels throughout the analysis. Cells appearing healthy in terms of size and shape were hand-traced in the phase channel before quantification of area, mean fluorescence, and integrated density in the fluorescence channel. Additionally, three circular regions of interest (ROIs) were drawn around each selected cell to capture background fluorescence levels. The CTCF was then computed using the formula: CTCF = integrated density – (area of cell × mean background fluorescence) (Fitzpatrick, 2014). This method was replicated across three or four independent experiments (N), with each group comprising 15 - 30 cells.

Data are displayed as the mean ± standard deviation (S.D.) on the graphs and the 95% confidence intervals (CI) are reported in the tables and text. A two-tailed unpaired t-test was used to compare two data sets, while analysis between three or more groups was accomplished via a one-way ANOVA with Dunnett’s multiple comparison test. All statistical analysis was run through GraphPad Prism software.

## 3 Results

### 3.1 The RVG peptide preferentially inhibits the human α7 subtype in comparison to heteromeric nAChRs

Rabies-infected animals greatly modify their behavior. These behavioral modifications involve areas of the CNS that express specific nAChR subtypes, namely α7, α3β2, α3β4, β3α6β2α4β2, α6β2β3, α4β2α5, and α4β2. To determine if the neurotoxin-like motif of the RVG could interact differentially with these nAChR subtypes and alter ACh-induced responses, we generated RVG peptide concentration-response curves of different nAChR subtypes.

**Figure 1:**
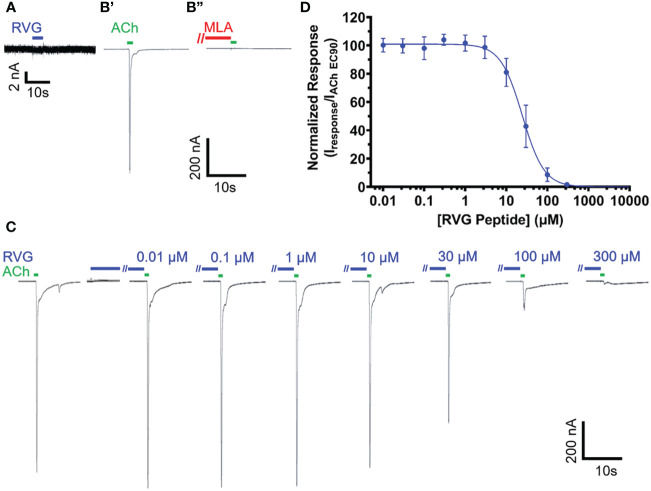
The RVG peptide potently antagonizes α7 nAChRs. RVG responses on either un-injected **(A)** or α7 nAChR-expressing **(B–D)** *Xenopus laevis* oocyte currents were recorded using TEVC electrophysiology. **(A)** Application of 100 μM RVG peptide for 2s (blue drug application bar) to an un-injected oocyte failed to elicit a response (N = 3, n = 3). A lack of evoked response was also observed at lower RVG peptide concentrations. **(B)** Example trace showing a control 1s ACh (EC_90_, 1.26 mM) (green drug application bar) α7 nAChR mediated response (B’) (green). Following a 1.5min wash, 30s pre-application of 10 nM MLA (red bar) prevented α7 nAChR activation via ACh (EC_90_, 1.26 mM) stimulation (B”) (N=1, n = 3). **(C)** α7 nAChRs were initially activated with ACh (EC_90_, 1.26 mM, green). To test for RVG peptide (blue) actions on α7 nAChRs, the RVG peptide (0.01 - 300 μM) was pre-applied for 30s, followed by a 1s ACh (EC_90_, 1.26 mM) stimulation. Importantly, as seen in the compiled RVG peptide (0.01 - 300 μM) responses, no agonist responses were observed at any tested RVG peptide concentration (first 7s shown). **(D)** Concentration-response profile of RVG peptide antagonized α7 nAChR ACh-evoked responses. The IC_50_ and n_H_ values are reported in **Table 2**. Points are the mean ± S.D. (N = 6, n = 6).

**Figure 2:**
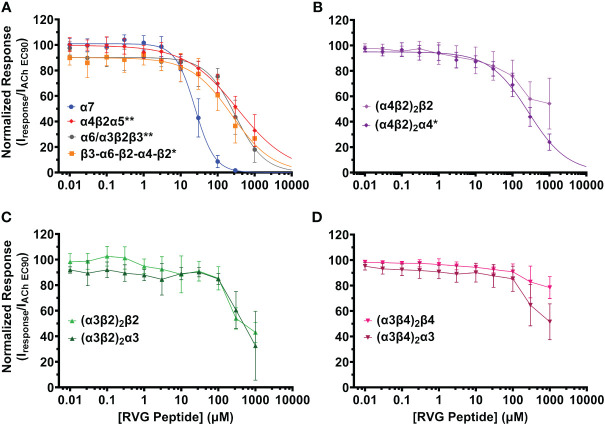
The RVG peptide selectively inhibits the α7 nAChR subtype with comparatively high apparent potency. nAChR-expressing *Xenopus laevis* oocytes were pre-exposed to increasing concentrations of the RVG peptide for 30s, followed by 1s stimulation with the subtype specific ACh EC_90_ (α4β2α5 20 µM; concatenated β3-α6-β2-α4-β2 40 µM; (α4β2)_2_β2 100 µM; (α4β2)_2_α4, α6/α3β2β3, (α3β2)_2_β2 316 µM; (α3β4)_2_β4 400 µM; (α3β4)_2_α3 800 µM, α7 1.26 mM, (α3β2)_2_α3 3.16 mM). **(A)** The α7 subtype was maximally inhibited by the RVG peptide while other isoforms lacking nAChR subtypes showed moderate inhibition (α7 nAChR data replicated from **Figure 1** to facilitate comparison). **(B)** The RVG peptide inhibited the (α4β2)_2_α4 isoform more than the (α4β2)_2_β2 isoform. **(C)** The α3β2 isoforms were similarly inhibited by the RVG peptide. **(D)** The (α3β4)_2_β4 isoform was minimally inhibited by the RVG peptide, while the (α3β4)_2_α3 isoform was inhibited to a greater extent. IC_50_, n_H,_ and p-values are reported in **Table 2**. Points are the mean ± S.D. (N = 3 - 6, n = 3 - 7).

Due to the sequence similarities of the RVG neurotoxin-like motif and loop 2 of αBTX (**Table 1**), we initially investigated the effect of the RVG peptide on human α7 nAChRs. Application of the RVG peptide to blank oocytes evoked no current (**Figure 1A**). When prompting the expression of the α7 nAChR, we accurately detected changes in receptor activity as we measured α7 nAChR activation with ACh (EC_90_), and inhibition using the established antagonist MLA (10 nM) (**Figure 1B**). The RVG peptide when applied alone did not produce agonist responses in our concentration range for any of the tested nAChRs (**Figure 1C** for α7 nAChR, and data not shown for heteromeric nAChR subtypes). The RVG peptide fully inhibited α7 nAChR mediated ACh responses in a concentration-dependent manner, with a more modest effect on all tested heteromeric nAChR subtypes, (**Figures 1C, D**, **2**). To determine the RVG peptide’s apparent potency, antagonized peak amplitude currents were measured and normalized to the ACh EC_90_ evoked response. The RVG peptide inhibited α7 nAChRs with a potency of 25 μM and fully inhibited (98%) the ACh-induced response with the application of 300 μM of the RVG peptide (**Figure 1D**, **Table 2**).

**Table 2:** Quantified data for RVG peptide concentration-response relations of ACh EC_90_ stimulated *Xenopus laevis* oocytes expressing the specified nAChR subtype.

nAChR Subtype	N (n)	IC_50_ (μM) (95% CI)	N_H_ (95% CI)	% Max Inhibition (95% CI)
α7	6 (6)	25 (22 – 28)	-1.6 (-1.4 to -2.0)	98 (97 – 99)
β3-α6-β2-α4-β2	5 (7)	240* (180 – 320)	-0.79 (-0.62 to -1.0)	73 (67 – 80)
α6/α3β2β3	5 (5)	300** (230 – 370)	-1.1 (-0.83 to -1.5)	82 (70 – 94)
α4β2α5	6 (7)	340** (280 – 430)	-0.62 (-0.52 to -0.73)	67 (52 – 83)
(α4β2)_2_β2	6 (6)	N.D.	N.D.	46 (21 – 71)
(α4β2)_2_α4	3 (3)	270* (220 – 340)	-0.82 (-0.66 to -1.0)	76 (60 – 92)
(α3β2)_2_β2	5 (6)	N.D.	N.D.	57 (44 – 70)
(α3β2)_2_α3	6 (7)	N.D.	N.D.	67 (25 – 110)
(α3β4)_2_β4	5 (6)	N.D.	N.D.	22 (11 – 32)
(α3β4)_2_α3	5 (5)	N.D.	N.D.	49 (31 – 66)

Comparison of α7 nAChR RVG peptide apparent potency was performed for those subtypes that had a concentration-response curve fit r^2^ value above 0.8: α4β2α5 **p = 0.0025, α6/α3β2β3 **p = 0.0078, (α4β2)_2_α4 *p = 0.0319, and β3-α6-β2-α4-β2 *p = 0.0149 (One-way ANOVA with a Dunnett’s multiple comparison’s test). Several nAChR subtypes’ concentration-response data could not be fit to a sigmoidal curve (r^2^ < 0.8), thus the RVG peptide potencies for these subtypes were not determined (N.D.).

The RVG peptide demonstrated inhibition of all tested heteromeric nAChR subtypes and their associated isoforms (**Figure 2**). The RVG peptide significantly inhibited the α4β2α5, α6/α3β2β3, (α4β2)_2_α4, and β3-α6-β2-α4-β2 subtypes (IC_50_’s 340 μM, 300 μM, 270 μM, and 240 μM, respectively), but the inhibition was less potent than α7 nAChRs (25 μM) (**Figure 2A**, **Table 2**). The ACh-induced responses of the (α4β2)_2_β2, α3β2, and α3β4 nAChR isoforms were also inhibited by the RVG peptide, but the extrapolated inhibition curves (r^2^ < 0.8) were associated with high error due to the smaller effects of the RVG peptide in the tested concentration range and thus were not included in statistical comparison of potencies (**Figures 2B–D**, **Table 2**).

**Figure 3:**
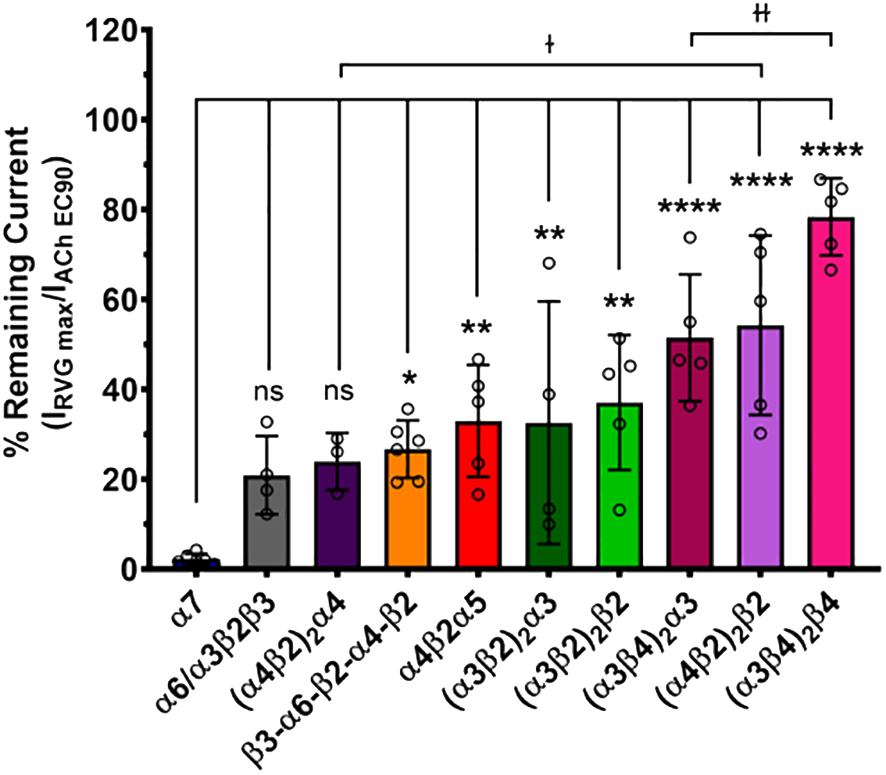
The α7 nAChR ACh-induced response was fully inhibited by 300 μM of the RVG peptide. The percent of ACh-induced current remaining post-RVG peptide exposure was determined for all tested nAChRs by performing concentration-response curves. The α7 nAChR was maximally inhibited by 300 μM of the RVG peptide, while the other subtypes were inhibited by varying amounts with 1000 μM RVG peptide. Significant changes in RVG peptide-induced inhibition are in reference to the α7 nAChR, and are indicated by the following statistical levels: β3-α6-β2-α4-β2 *p = 0.025, α4β2α5 **p = 0.0046, (α3β2)_2_α3 **p = 0.0099, (α3β2)_2_β2 **p = 0.0011, (α4β2)_2_β2 ****p < 0.0001, (α3β4)_2_α3 ****p < 0.0001, (α3β4)_2_β4 ****p < 0.0001 (One-way ANOVA with Dunnett’s *post-hoc* test). Two-tailed unpaired t-tests were performed on the α4β2, α3β4, and α3β2 subtypes to assess differences between isoforms for each subtype. The (α4β2)_2_α4 was inhibited significantly more than the (α4β2)_2_β2 isoform (^ɫ^p = 0.0476). Similarly, the (α3β4)_2_α3 was inhibited greater than its (α3β4)_2_β4 counterpart (^ɫɫ^p = 0.0066). Values are mean ± S.D. with individual oocytes shown as symbols (N = 3 - 5, n = 4 - 6).

The maximum amount of inhibition of the ACh-induced responses by the RVG peptide varied between the nAChR subtypes. The α7 subtype was maximally and fully inhibited with 300 μM of the RVG peptide (**Figure 3**, **Table 2**). At the highest RVG peptide concentration applied (1 mM) the heteromeric subtypes displayed varying amounts of inhibition with α6/α3β2β3 being inhibited the most after the α7 nAChR, and the (α3β4)_2_β4 subtype being inhibited the least (**Figure 3**, **Table 2**). The rank order of RVG peptide inhibition is as follows: α7 > α6/α3β2β3 > (α4β2)_2_α4 > β3-α6-β2-α4-β2* > (α3β2)_2_α3** > α4β2α5** > (α3β2)_2_β2** > (α3β4)_2_α3**** > (α4β2)_2_β2**** > (α3β4)_2_β4**** (listed from greatest to the least amount of inhibition; stars indicate the level of significance determined via One-way ANOVA with Dunnett’s *post-hoc* analysis, as described in the legend for **Figure 3**). For the α4β2 and α3β4 heteromeric nAChRs, the isoforms that had an additional α subunit were inhibited significantly more by the RVG peptide than their counterparts, suggesting a role in the α/α interface for enhanced inhibition (**Figure 3**). Together, our findings demonstrate that the RVG peptide inhibits ACh-induced responses of the explored nAChR subtypes, but shows a high preference for the α7 subtype.

### 3.2 RVG peptide is an α7 nAChR competitive antagonist

**Figure 4:**
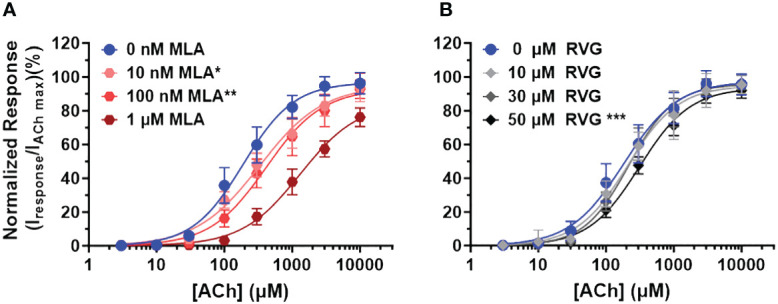
The RVG peptide competes with ACh for binding to the α7 nAChR. α7 nAChR-expressing *Xenopus laevis* oocytes were co-exposed to increasing concentrations of ACh and either the known competitive antagonists MLA or the RVG peptide. Two ACh Imax (10 mM) applications without MLA or RVG peptide were performed following the co-application responses. All responses were normalized to the second application of 10 mM ACh. **(A)** Co-application of 10 nM or 100 nM MLA significantly reduced ACh apparent potency without altering the maximal response. Sufficient ACh could not be applied to outcompete 1 µM MLA as ACh will block α7 nAChRs at concentrations greater than 10 mM. **(B)** The RVG peptide when co-applied with increasing concentrations of ACh also shifted the response curve to the right, reaching statistical significance with 50 µM. The maximal response was not altered at any RVG concentration tested. Data points are mean ± S.D. (N = 3 - 4, n = 9 - 14). IC_50_, n_H,_ and p-values are reported in **Table 3**.

To explore the mechanism of RVG peptide inhibition on α7 nAChRs, we performed a competitive antagonist assay. 0 nM, 10 nM, 100 nM, or 1 µM MLA were co-applied (a distinctly different method from the pre-application drug treatment used in **Figures 1**, **2**) with increasing concentrations of ACh (3 - 10,000 µM) (**Figure 4A**, **Table 3**). As anticipated for a competitive antagonist, higher concentrations of MLA significantly shifted the apparent potency of the ACh curve to the right without altering efficacy. At 1 µM MLA, we were not able to reach the maximal response by increasing the ACh concentration as ACh will channel block at concentrations above 10,000 µM. We then used the same protocol to examine the possible mechanism of inhibition using the RVG peptide (**Figure 4B**, **Table 3**). Similar to MLA, with increasing concentrations of the RVG peptide (10, 30, or 50 µM) a rightward shift in the ACh apparent potency alongside no change in the efficacy was observed. Using 50 µM of RVG peptide, the ACh apparent potency was significantly shifted from 182 µM (CI 156, 214 µM) (ACh only) to 310 µM (CI 280, 335 µM) (**Figure 4B**, **Table 3**). These results show that the RVG peptide is an α7 nAChR competitive antagonist.

**Table 3:** Calculated parameters for MLA and RVG peptide competitive antagonist ACh concentration-response profiles.

Drug Treatment	N (n)	IC_50_ (μM) (95% CI)	N_H_ (95% CI)
None	4 (14)	182 (156 - 215)	1.1 (1.0 - 1.2)
10 nM MLA	4 (13)	330* (272 - 411)	0.9 (0.8 - 1.1)
100 nM MLA	4 (14)	412** (342 - 508)	1.1 (0.9 - 1.2)
1 µM MLA	3 (9)	N.D.	N.D.
10 µM RVG	4 (12)	208 (175 - 250)	1.2 (1.0 - 1.4)
30 µM RVG	4 (12)	217 (195 - 245)	1.3 (1.2 - 1.5)
50 µM RVG	4 (11)	310*** (280 - 335)	1.2 (1.1 - 1.3)

Data was fit with monophasic curves as determined by a sum of squares F-test. MLA-treated ACh concentration-response profiles resulted in significantly reduced ACh potencies compared to the untreated α7 nAChR ACh apparent potency (One-way ANOVA with Dunnett’s multiple comparison test F (2, 8) = 12.25, 10 nM *p = 0.0233 and 100 nM **p = 0.0021). Co-application of 50 µM of the RVG peptide also shifted the ACh apparent potency to the right without altering efficacy, demonstrating a competitive antagonist mechanism (One-way ANOVA with Dunnett’s multiple comparison test F (3, 12) = 14.28, ***p = 0.0001).

### 3.3 Alanine substitutions of key residues in the RVG peptide alter peptide activity on α7 nAChRs

To determine RVG peptide residues that are important for interaction with the α7 nAChR subtype, we compared the RVG peptide sequence to αBTX and other neurotoxins (**Table 1**, residues bolded are highly conserved). Based on the most recent α7 nAChR cryogenic electron microscopy (cryo-EM) structure (PDB 7KOO), αBTX residues F32, S35, and R36 (RVG equivalent F192, S195, and R196) are important for binding to the resting state (Noviello et al., 2021). Interestingly, the αBTX valine (V) 39 residue (sequence alignment positioning equivalent to RVG R199) is positioned to bind to the α7 subtype via a cation-π interaction, but cation-π interactions are not possible with V. However, in the RVG neurotoxin-like region, an R is found at this position (R199) and could potentially form a cation-π interaction with the α7 nAChR. This R is also conserved among other neurotoxins (**Table 1**). To determine if corresponding residues are important in the binding of the RVG peptide as in αBTX, we made individual A substitutions at positions F192, S195, R196, or R199 in the RVG peptide and performed concentration-response experiments. An A substitution was chosen as it replaces the side chain at the β-carbon for a chemically inert methyl group allowing for the detection of functionally important positions in the RVG neurotoxin-like motif (Bromberg and Rost, 2008).

**Figure 5:**
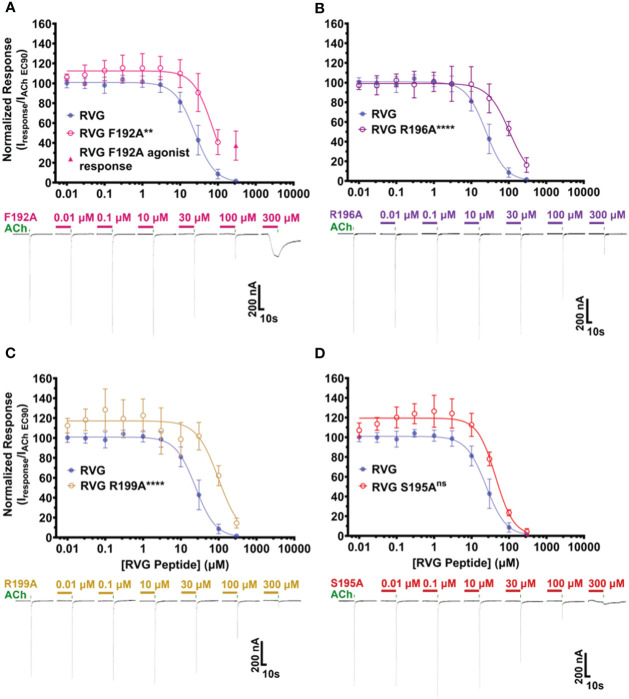
RVG F192A, R196A, and R199A peptides inhibit the function of α7 nAChRs distinctly from the RVG peptide. α7 nAChR-expressing *Xenopus laevis* oocytes were pre-exposed to 30s of increasing concentrations of each altered RVG peptide, and inhibition of the ACh EC_90_ response was measured. The α7 nAChR RVG peptide inhibition curve from **Figure 1** is shown on each panel to facilitate comparison to the altered peptides. **(A)** The RVG F192A peptide concentration-response curve was shifted to the right relative to the RVG peptide. Application of 300 μM RVG F192A peptide evoked an agonist response. **(B)** The RVG R196A and **(C)** RVG R199A peptides showed reduced apparent potency on α7 nAChRs compared to the RVG peptide. **(D)** The RVG S195A peptide exhibited a similar concentration-response profile to the RVG peptide. IC_50_, n_H_, and p-values are reported in **Table 4**. Points are mean ± S.D. (N = 3 - 5, n = 3 - 6).

The first RVG peptide residue examined was F192. In comparison to the RVG peptide, the F192A peptide significantly reduced the peptide’s apparent potency (RVG peptide 25 μM vs. RVG F192A 71 μM) (**Figure 5A**, **Table 4**). Surprisingly, with the application of 300 μM of RVG F192A peptide in the absence of an agonist, we observed a robust agonist response. This agonist effect was not present with the RVG peptide or any of the other peptides examined. The RVG F192A peptide also inhibited the α7 nAChR significantly less than the RVG peptide (59% vs. 98%) (**Figure 5A**, **Table 4**).

**Table 4:** Alaine substituted RVG peptide concentration-response relations of ACh EC_90_ stimulated *Xenopus laevis* oocytes expressing the α7 nAChR subtype.

Peptide	N (n)	IC_50_ (μM) (95% CI)	N_H_ (95% CI)	% Max Inhibition of ACh EC_90_ (95% CI)
RVG	6 (6)	25 (22 – 28)	-1.6 (-1.4 to -2.0)	98 (97 – 99)
RVG F192A	4 (4)	71** (54 – 89)	-1.7 (-1.0 to -2.4)	59**** (39 – 79)
RVG R196A	5 (5)	110**** (86 – 130)	-1.5 (-1.1 to -1.9)	84* (77 – 91)
RVG R199A	4 (4)	100**** (65 – 136)	-1.5 (-0.74 to -2.2)	86* (73 – 98)
RVG S195A	3 (3)	44^ns^ (34 – 54)	-1.8 (-1.2 to -2.4)	96^ns^ (88 – 100)

Comparisons of RVG peptide apparent potency to altered peptides on α7 nAChRs were performed: RVG F192A **p = 0.0019, RVG R196A ****p < 0.0001, RVG R199A ****p < 0.0001 and S195A ^ns^p = 0.4032 (One-way ANOVA with Dunnett’s multiple comparison test). The percent maximum inhibition for each mutant peptide was compared to the RVG peptide using α7 nAChRs: RVG F192A ****p < 0.0001, RVG R196A *p = 0.0140, RVG R199A *p = 0.0481, and RVG S195A ^ns^p = 0.9878 (One-way ANOVA with Dunnett’s multiple comparison test).

Both the RVG R196A and RVG 199A peptides inhibited ACh-induced responses in a concentration-dependent manner but were significantly less potent on α7 nAChRs than the RVG peptide (RVG R196A 110 μM and RVG R199A 100 μM) (**Figures 5B, C**, **Table 4**). RVG R196A and R199A peptides inhibited the α7 nAChR ACh-induced responses significantly less (84% and 86%, respectively) than the RVG peptide (**Figures 5B, C**, **Table 4**). Importantly, the RVG S195A substitution showed no effect in altering the RVG peptide apparent potency (44 μM) or the amount of inhibition (96%) in comparison to the unaltered RVG peptide (**Figure 5D**, **Table 4**). Surprisingly, the RVG F192A, R199A, and S195 peptides were all observed to very slightly enhance ACh mediated currents at low peptide concentrations (0.01 - 3 μM). Our results demonstrate that RVG peptide residue’s F192 and R196 are important for RVG interactions with the α7 nAChR, similar to αBTX. In a point of sequence difference between αBTX and RVG, changing residue R199 dramatically reduced RVG peptide potency demonstrating that the RVG peptide does utilize novel residues to interact with α7 nAChRs. This is further supported by our findings that residue S195 is not critical for the peptide’s interactions as the A substitution induced no change in RVG peptide apparent potency or inhibition.

### 3.4 RVG ectodomain potently inhibits α7 nAChR function

**Figure 6:**
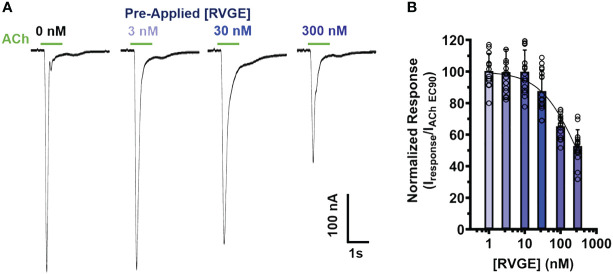
RVGE robustly inhibits α7 nAChRs response to ACh with nanomolar apparent potency. **(A)** α7 nAChR-expressing oocytes were bath exposed to a single concentration of RVGE (1 - 300 nM) for 5min before EC_90_ (1.26 mM) ACh application (1s). Example traces are shown. **(B)** Increasing concentrations of RVGE inhibited ACh-induced responses potently. Data were normalized to the control response. Non-linear regression curve fit to RVGE data with an IC_50_ of 295 nM [CI 233, 399]. Data are mean ± S.D. (N = 2 - 3, n = 12 - 22).

To determine if our α7 nAChR RVG peptide findings could be recapitulated using the full RVG ectodomain (RVGE), we determined the apparent potency of the RVGE on human α7 nAChRs by performing concentration-response electrophysiology experiments. Each α7 nAChR-expressing oocyte was incubated with a single RVGE concentration (1 - 300 nM) for 5min in a static bath, followed by a 1s ACh EC_90_ stimulation (**Figure 6A**). The resulting ACh-induced responses were robustly inhibited with an RVGE apparent potency of 295 nM (CI 233, 399 nM) (**Figure 6B**). Our findings generated with RVGE are consistent with those obtained with the RVG peptide. The improved RVGE apparent potency is likely due to the increased structural restraints of RVGE compared to the RVG peptide.

### 3.5 Fluorescently tagged RVG peptide targets α7 nAChRs in cultured mammalian neuronal-like cells

We next wanted to determine if the RVG peptide interacted with mammalian cells expressing the α7 nAChR. We chose N2a cells as the cell line is derived from mouse neuroblasts and does not robustly express the α7 subtype endogenously. To ensure our α7 nAChR DNA transient transfection procedures resulted in robust plasma membrane expression, we transfected N2a cells with α7-pHuji plasmid DNA (**Supplementary Figure 2**). pHuji, a pH-sensitive fluorophore, was C-terminally tagged to the α7 subunit and exposed to the extracellular environment (Shen et al., 2014). Cells were imaged in a pH 7.4 buffer, locations were saved, and re-imaged following a pH 5.0 buffer change (**Supplementary Figure 2A**). As pHuji fluorescence is quenched at pH 5.0, any captured fluorescence at pH 5.0 (**Supplementary Figure 2B**) is due to α7-pHuji nAChRs located intracellularly and not on the plasma membrane (**Supplementary Figure 2C**). Using these image series, the location of α7-pHuji nAChR expression could be verified to be largely on the plasma membrane (**Supplementary Figure 2D**).

**Figure 7:**
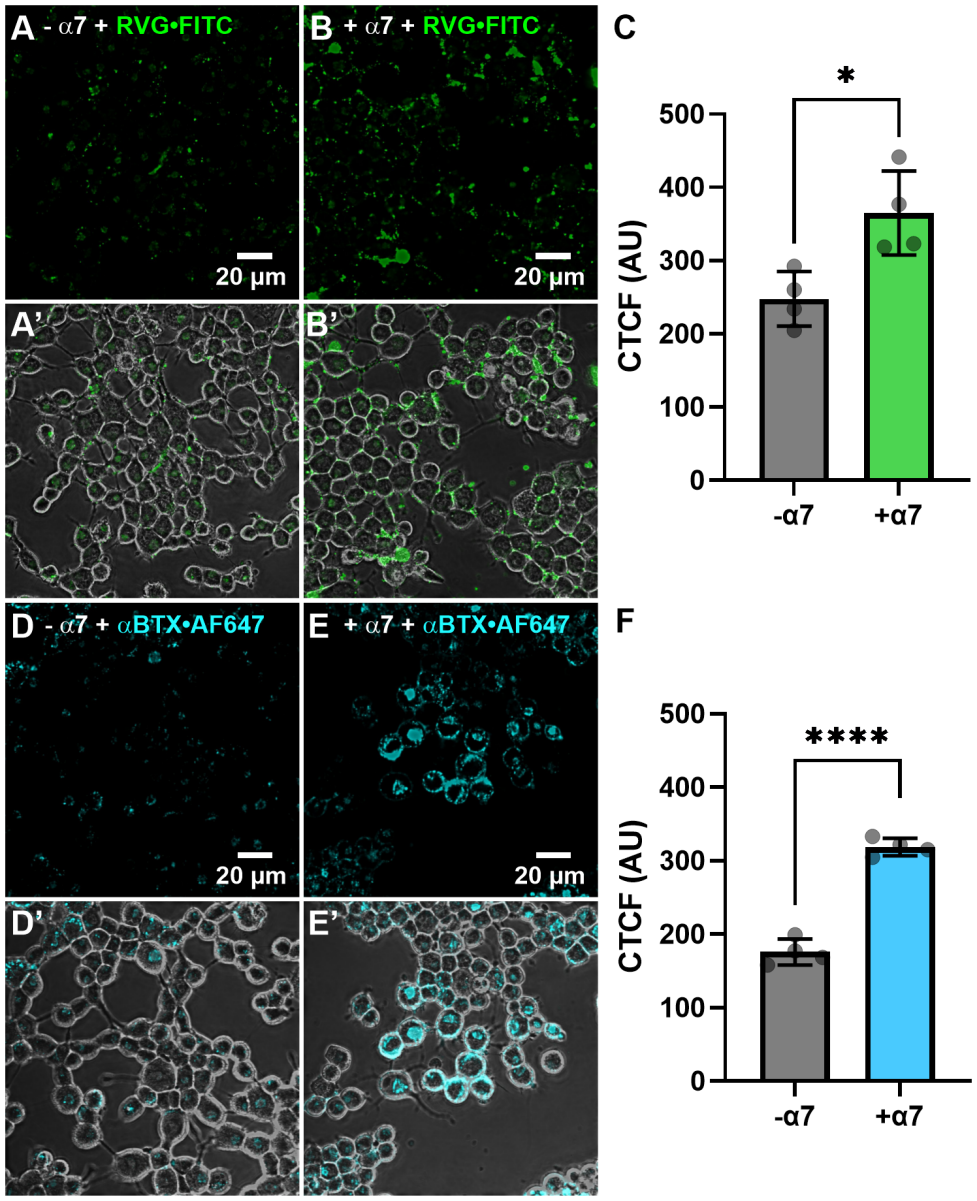
The RVG-FITC peptide favorably labels cells expressing the α7 nAChR. Non-transfected and α7 nAChR-transfected N2a cells were treated with 30 µM RVG-FITC or 80 nM αBTX-AF647 for 24hr prior to live-cell confocal imaging. **(A)** Example image of RVG-FITC labeling using non-transfected N2a cells. **(B)** RVG-FITC robustly labeled α7 nAChR-expressing N2a cells. **(A’, B’)** Same as A and B images with the addition of the phase channel. **(C)** CTCF analysis of RVG labeling of transfected and non-transfected N2a cells (*p = 0.0180, two-tailed unpaired T-test). **(D)** αBTX-AF647 staining of endogenous α7 nAChRs expressed in non-transfected N2a cells. **(E)** α7 nAChR-transfected N2a cells labeled with αBTX-AF647. **(D’, E’)** Same as D and E images with the addition of the phase channel. **(F)** CTCF analysis of αBTX-AF647 labeling of transfected and non-transfected N2a cells (****p < 0.0001, two-tailed unpaired t-test). Images shown are representative of four separate experiments per group. CTCF data are mean ± S.D. (N = 4, n = 60 – 120).

We initially wanted to determine if the RVG peptide tagged with the FITC fluorophore interacted with proteins endogenously expressed on the plasma membrane of N2a cells not transfected with α7 DNA. Thirty µM of the RVG-FITC peptide was applied for 24hr prior to performing live-cell confocal imaging. We observed modest RVG peptide labeling (250 ± 40 AU) (**Figures 7AA’**) and the fluorescence was significantly enhanced when applied to N2a cells transfected with human α7 nAChR DNA (370 ± 60 AU) (*p = 0.018, two-tailed unpaired t-test) (**Figures 7B, B’, C**). The RVG peptide appeared to be located inside the cells, which we confirmed using z-stacks (**Supplementary Figure 2**). As a control, we used αBTX labeled with the fluorophore AF647 (αBTX-AF647) to detect α7 nAChR expression. αBTX is highly selective for the α7 subtype of the neuronal nAChRs (Noviello et al., 2021). Using un-transfected cells, αBTX-AF647 labeling was very light, demonstrating that N2a cells express low levels of α7 nAChRs endogenously (180 ± 20 AU) (**Figures 7D, D’**). Testing αBTX-AF647 on α7 nAChR DNA-transfected cells, we observed a significant increase in fluorescence (320 ± 10 AU) (****p < 0.0001, two-tailed unpaired t-test) (**Figures 7E, E’, F**). Interestingly, when comparing the RVG-FITC peptide (**Figure 7A**) and the α7 subtype-selective αBTX-AF647 labeling (**Figure 7D**), there is notably enhanced staining with RVG-FITC in both the non-transfected and α7 nAChR-transfected groups (*p = 0.023, two-tailed unpaired t-test). These results confirm our electrophysiology observations and literature (Schnell et al., 2010), that RVG has multiple cellular targets, including a high preference for the α7 nAChR.

## 4 Discussion

Virus-receptor interactions are most often studied to understand virus entry into host cells. Here we expand our understanding of these interactions to include modulation of receptor function. We identify that the neurotoxin-like motif found in the RVG interacts with nAChR subtypes to antagonize receptor signaling. The RVG neurotoxin-like peptide inhibits all the examined nAChR subtypes with varying degrees of specificity. The α7 subtype was inhibited the most (98%) using 300 μM of the RVG peptide, with high apparent potency (25 μM) compared to the other tested nAChRs. We determined that like MLA, the RVG peptide can compete with ACh for the orthosteric site and is thus a competitive antagonist. Identified by A substitutions, residues F192, R196, and R199 are important for manipulating RVG peptide potency for the α7 subtype. We expanded our discoveries to encompass the complete ectodomain, illustrating that RVGE can similarly act as an α7 nAChR antagonist, exhibiting nanomolar potency. To begin translating our work to mammals, we found that the RVG peptide targets α7 nAChRs expressed on the plasma membrane of neuronal-like cells. Our findings provide a detailed analysis of the RVG selectivity for specific nAChR subtypes and characterize the neurotoxin-like region’s ability to modify α7 nAChR function.

### 4.1 RVG peptide and heteromeric nAChRs

Inhibition of neuronal nAChRs could have significant consequences for rabies virus pathogenesis, including, but not limited to, behavior modifications. Many pathogens specifically modulate host behavior to increase transmission to a new host (Poulin, 1995; Vyas et al., 2007; Cezilly et al., 2010; Adamo, 2013; Knight, 2013). The rabies virus provides a traceable model to probe these modifications as it has only five genes and a much simpler life cycle than eukaryotic pathogens. Many of these pathogen-driven behavior manipulations, however, are not well understood at the cellular and molecular levels. The RVG peptide modified the behavior of *C. elegans* and mice (Hueffer et al., 2017). Specifically, the RVG peptide inhibited the frequency of nAChR-meditated pharyngeal pumping in *C. elegans*, and induced hyperactivity (a rabies-associated behavior) after intraventricular administration into the CNS of mice. In efforts to identify the cellular host target, a population of mixed α4β2 nAChR isoforms was functionally inhibited by both the neurotoxin-like region of RVG and the ectodomain of RVG (Hueffer et al., 2017).

Our findings are in line with those of Hueffer et al., 2017. This previous work identified that the RVG peptide showed moderate α4β2 nAChR (mixed isoform population) apparent potency (extrapolated IC_50_ 185 - 314 μM (95% CI)), which compares well with our findings ((α4β2)_2_α4 220 - 340 μM (95% CI)) (Hueffer et al., 2017). In our expanded study, the activity of the RVG neurotoxin-like peptide on other nAChR subtypes and isoforms shows that the peptide inhibits ACh-induced responses with varying efficiencies. The two isoforms of α4β2, (α4β2)_2_α4, and (α4β2)_2_β2, differ three- to four-fold in their susceptibility to inhibition by the RVG peptides, with the greatest inhibition effects on the (α4β2)_2_α4 isoform. Interestingly, we noted that of the tested heteromeric nAChRs, isoforms that contained a third α subunit were inhibited more than their counterparts with only two α subunits. These data suggest that α/α interfaces may be preferential for RVG peptide interactions, which is further supported by our findings of strongest inhibition on the α7 nAChR subtype, which has five α/α interfaces. As ligand apparent potency is enhanced by the presence of multiple binding sites (Whiteaker et al., 2007), the enhanced apparent potency of the peptide for the α7 subtype alludes that this may be the case for the RVG peptide. Our data confirming that the peptide competes for the orthosteric site supports the presence of multiple binding sites, as ACh has five binding sites on α7 nAChRs.

Our work further highlights the promiscuity of the RVG neurotoxin-like motif and its interactions with nAChR subtypes. During rabies infection, the host has altered locomotor behavior, including increased ranging distance, hyperactivity, and ataxia (Rupprecht et al., 2002; Jackson, 2016b). The α4β2 and α7 subtypes are the most common nAChRs in the CNS and are linked to hyperactivity, aggression, and anxiety (Ross et al., 2000; Champtiaux et al., 2003; King et al., 2004; Lewis et al., 2015; Picciotto et al., 2015), behavioral features also common to rabies infection (Jackson, 2016b). These subtypes are also present in the brainstem (Champtiaux et al., 2003; Hogg et al., 2003; Gotti et al., 2005), which has integrative functions that are altered with rabies infection, including controlling the cardiovascular system, respiratory control, pain sensitivity, alertness, awareness, and consciousness. While the α7 nAChR ACh response was fully inhibited, the α6/α3β2β3, (α4β2)_2_α4, and β3-α6-β2-α4-β2 subtype ACh responses were inhibited 79%, 76% and 73%, respectively, albeit with a reduction in RVG apparent potency. The β3α6β2α4β2, α6β2β3, and α4β2α5 nAChR subtypes are enriched in the nigrostriatal dopaminergic system, an important pathway in regulating locomotor behavior (Luetje, 2004; Salminen et al., 2004; Grady et al., 2007; Gotti et al., 2010; Quik et al., 2011). The α3β2 and α3β4 subtypes were inhibited by RVG at only very high concentrations and thus may not be involved in host viral infection.

### 4.2 RVG and α7 nAChRs

The RVG peptide inhibits the α7 nAChR subtype more potently and fully in comparison to the other tested heteromeric subtypes. Using a soluble rabies ectodomain, we demonstrated that the ectodomain antagonizes α7 nAChRs with an improved apparent potency of 295 nM in comparison to the RVG peptide (25 µM). The improved potency was expected as the RVGE likely has enhanced structural restraints compared to the RVG peptide. This aligns with previous findings that the full ectodomain more strongly inhibits α4β2 nAChRs compared to the RVG peptide (Hueffer et al., 2017).

Clues identifying important RVG peptide residues involved in nAChR interactions can be seen by comparison with several neurotoxin protein sequences. Loop II of αBTX and α-cobratoxin have relatively high sequence homologies to the RVG neurotoxin-like motif (**Table 1**). Many snake neurotoxins function as potent nAChR subtype-selective antagonists (Barber et al., 2013). A previous study has shown that RVG neurotoxin-like peptides, similar to snake venom toxins, bind to the *Torpedo* electric organ ACh receptor. This interaction is inhibited by AChR agonists and antagonists, suggesting that the RVG peptides bind close to or at the ACh binding site of the receptor (Lentz et al., 1987). We have confirmed these speculations and have demonstrated that the RVG peptide is a competitive antagonist, interacting at the orthosteric site.

Using circular dichroism spectroscopy, a 20-residue peptide of the king cobra loop II and a 29-residue RVG peptide containing the neurotoxin-like motif were shown to be conformationally similar, containing mostly beta-sheet structure (Donnelly-Roberts and Lentz, 1991). Several key α-neurotoxin residues are important for determining affinity (Lentz, 1991; Chiappinelli et al., 1996; Antil-Delbeke et al., 2000; Fruchart-Gaillard et al., 2002; Bourne et al., 2005; Huang et al., 2013). X-ray crystallography and cryo-EM structures have revealed great detail into the importance of αBTX residues F32 and R33, along with α-cobratoxin R36 for facilitating interactions at the nAChR orthosteric binding site (Antil et al., 1999; Antil-Delbeke et al., 2000; Servent et al., 2000; Marinou and Tzartos, 2003; Rahman et al., 2020; Noviello et al., 2021). Based on the free-form RVG crystal structure (Yang et al., 2020), the neurotoxin-like motif is located on the surface, solvent-exposed, and is positioned favorably to interact with cellular targets, including nAChRs (Lian et al., 2022). Early conformational studies on the RVG asparagine (N)194-S195-R196-glycine (G)197 tetrapeptide sequence from the neurotoxin-like region determined this as an essential part of the binding site for nAChRs (Rustici et al., 1993). Importantly, the side chains of N194 and R196 may mimic the ACh structure.

In addition to conservation among α-neurotoxins, residues 192, 195, 196, and 199 are also highly conserved among RVG isolates (Hueffer et al., 2017). When comparing 2,600 RVG sequences, residue 192 is an F in all but three sequences, and position 195 is an S in all isolates. Residues 196 and 199 show higher levels of polymorphism with all but one of the 2,600 isolates having a positively charged amino acid residue (R or lysine (K)) at these two positions. Alaine substitutions at F192, R196, and R199 in our study demonstrated that these residues, as predicted by the α-neurotoxin structure studies and RVG isolate sequence comparisons described immediately above, are important for RVG peptide interactions with α7 nAChRs. As expected, residues F192 and R196 are important in determining the apparent potency of the RVG peptide for the α7 nAChR, as A substitutions significantly reduced the modified RVG peptide potency. Unexpectedly, at the highest concentration (300 μM) of the RVG F192A peptide, we observed an agonist response. This suggests that residue F192 is interacting at the orthosteric site and/or is important in stabilizing or facilitating receptor closed or open states, or may function as a partial, low-efficacy agonist.

Residue RVG R199 was also shown to be important in determining RVG peptide apparent potency on α7 nAChRs. In αBTX, this residue is a V (V39), which has very distinct physiochemical properties from R. Our observation that an A substitution at position 199 reduced RVG peptide apparent potency on α7 nAChRs suggests that an R at this position is important for RVG’s functional α7 nAChR potency. The role of residue RVG S195 was explored because the αBTX equivalent residue S25 interacts via an H-bond with an N-linked glycan from α7 subunit residue N110 on the complementary subunit, and electrostatically with Q116 on the principal subunit (Noviello et al., 2021). The RVG S195A peptide did not affect α7 nAChR apparent potency, suggesting that this residue is not essential in mediating nAChR interactions. These findings demonstrate that there are similarities in how the RVG peptide and α-neurotoxins interact with the α7 nAChR, but the RVG peptide also utilizes (or not as in the case of S195) unique residues to antagonize receptor function.

### 4.3 Physiological implications

The rabies virus targets several host receptors in addition to the muscle and α4β2 nAChR subtypes, and we have now provided functional and cell culture data demonstrating that the α7 nAChR is also an RVG target in mammalian cells with a neuronal-like phenotype. Consistent with the observations that N2a cells endogenously express other rabies virus-host targets, including the neural cell adhesion molecule (Bhat and Silberberg, 1988; Kadmon et al., 1990), p75 neurotrophin receptor (Bai et al., 2008), metabotropic glutamate receptor subtype 2 (Wang et al., 2018) and integrin β1 (Shuai et al., 2020), the RVG-FITC peptide lightly stained N2a cells not transfected with α7 nAChR DNA. Importantly, the RVG peptide binding was greatly enhanced when the N2a cells robustly expressed α7 nAChRs.

RVG-driven nAChR functional inhibition will likely have important physiological implications when considering hosts infected with rabies. Using a fluorescence micro‐optical sectioning tomography (fMOST) system, the 3D spatial distribution of rabies virus in intact mice brains has been elegantly imaged (Zhang et al., 2022). The infected regions included areas enriched with α7 nAChRs, specifically the motor cortex, amygdala, and hippocampus. While the rabies viral load in the human CNS during any stage of infection is poorly studied, using mice showing robust clinical signs of rabies and qt-PCR, the CNS viral titer has been estimated to be 1.6 - 8.3 x 10^7^ viral genomes per μg of total RNA, depending on the viral strain (Bertoune et al., 2017). It is worth noting that viral loads differ by brain regions, viral type, and stages of infection (Laothamatas et al., 2008; Zhang et al., 2022).

α7 and α4β2 nAChRs are expressed along axons, dendrites, and cell bodies, in addition to pre- and post-synaptic locations (Zarei et al., 1999; Jones and Wonnacott, 2004; Zhong et al., 2008). A soluble form of RVG, which lacks the C-terminal anchoring region of the glycoprotein, has been detected in the media of rabies-infected cultured cells (Morimoto et al., 1993; Thirapanmethee et al., 2005). As the hippocampal synaptic cleft (~20 nm (Schikorski and Stevens, 1997)) is too small to accommodate the rabies virion (60 x 180 nm (Rupprecht, 1996)), it is likely that soluble RVGs, if these are produced *in vivo*, target pre- and post-synaptic α7 nAChRs within the hippocampus (Fabian-Fine et al., 2001b). A lingering question remains if soluble RVGs are shed into the synaptic space.

The rabies virus is transmitted by animal bites, which is facilitated by increased host aggression induced by the virus (Fooks et al., 2017). Interestingly, inhibiting the activity of the α7 nAChR either via pharmacological means or by reducing expression of the α7 nAChR, as in the 15q13.3 microdeletion syndrome, animal aggression is augmented (Shinawi et al., 2009; Fejgin et al., 2014; Lewis et al., 2015; Gillentine et al., 2017; Lewis et al., 2018; Lewis and Picciotto, 2020). In contrast, activation of α7 nAChRs by agonists, including nicotine and GTS-21, have been proposed as mechanistic explanations for the ‘serenic’ effects of smoking (Lewis et al., 2015). RVG has also recently been shown to target the α7 nAChR and induce the cholinergic anti-inflammatory pathway in monocyte-derived macrophages (Embregts et al., 2021). In immune cells, the α7 subtype likely functions as a metabotropic, rather than as an ionotropic receptor, to activate different pathways (de Jonge et al., 2005; Kabbani et al., 2013; Papke, 2014; Kabbani and Nichols, 2018). For example, full agonist and positive allosteric modulators that increase α7 nAChR ion channel currents fail to reduce Tumor Necrosis Factor α (TNFα) production in microglial cells (Thomsen and Mikkelsen, 2012). In contrast, ligands that are very weak partial agonists or antagonists reduce lipopolysaccharide (LPS)-induction of TNFα. Here we have demonstrated that the RVG peptide and RVGE antagonized α7 nAChR function. It remains to be determined if RVG can prevent α7 nAChR-enabled pro-inflammatory responses as a mechanism to evade the immune system. While our work begins to demonstrate a possible neuronal nAChR-mediated mechanism in rabies-modified host behavior, animal behavior work using either α7 nAChR knockout animal models or pharmacologic tools, including α7 nAChR antagonists, is necessary to verify our speculation. Understanding the functional effects of RVG on specific nAChRs enhances our understanding of rabies pathogenesis in humans and presents new avenues for specific therapeutic approaches to treat rabies infections in humans.

## Data availability statement

The raw data supporting the conclusions of this article will be made available by the authors, without undue reservation.

## Ethics statement

The animal study was approved by the University of Alaska Fairbanks’ Institutional Animal Care and Use Committee. The study was conducted in accordance with the local legislation and institutional requirements.

## Author contributions

BO’B: Data curation, Formal analysis, Methodology, Writing – original draft, Writing – review & editing. ST: Data curation, Funding acquisition, Writing – review & editing. LW: Data curation, Formal analysis, Methodology, Visualization, Writing – review & editing. HD: Data curation, Methodology, Validation, Writing – review & editing. AB: Data curation, Formal analysis, Writing – review & editing.. KH: Conceptualization, Funding acquisition, Resources, Writing – review & editing. MW: Conceptualization, Data curation, Formal analysis, Funding acquisition, Investigation, Methodology, Project administration, Resources, Supervision, Validation, Visualization, Writing – original draft, Writing – review & editing.
