# Supplementary material for: The human alpha7 nicotinic acetylcholine receptor is a host target for the rabies virus glycoprotein
Source: Front Cell Infect Microbiol. 2024 May 21;14:1394713. doi: 10.3389/fcimb.2024.1394713 (PMC11148329; doi:10.3389/fcimb.2024.1394713)
Supplement: Supplementary file 1 [file DataSheet_1.docx]

Supplementary Material

# Supplementary Data

**1.1 Expression of nAChR subtypes and associated isoforms.**

To test for the potential effect of the RVG peptide on nAChR subtypes possibly involved in rabies infection, we expressed human nAChR subtypes in *Xenopus laevis* oocytes (see *Methods* and Supplemental Table 1). To ensure we had expression of each nAChR subtype and desired isoform, we determined the apparent potency via ACh concentration-response profiles for each nAChR preparation (Supplemental Figure 1 and Supplemental Table 1). The α7, α4β2α5, α6/α3β2β3, and β3-α6-β2-α4-β2 subtypes produced functional receptors that generated sigmoidal ACh concentration-response curves (Supplemental Figure 1A). Measured ACh apparent potency values are consistent with the literature [39, 50, 54-57, 104-107], with the α4β2α5, α6/α3β2β3, and β3-α6-β2-α4-β2 nAChR subtypes being the most sensitive to ACh (EC_50_’s 1.1 μM, 4.5 μM and 1.5 μM, respectively). The α7 subtype had the lowest ACh apparent potency (EC_50_ 140 μM) (Supplemental Table 1).

The isoforms of α4β2, α3β2, and α3β4 nAChRs were expressed by biasing the α : β cRNA injection ratio (see the *Methods* section and Supplemental Table 1 for details) [49, 58]. Each of these subtypes expresses in isoforms with high sensitivity (HS) or low sensitivity (LS) to agonists (HS (αβ)_2_β or LS (αβ)_2_α nAChRs, respectively). The (α4β2)_2_β2 HS-isoform produced receptors with high apparent potency to ACh (3.9 μM) (Supplemental Figure 1B, Supplemental Table 1). The (α4β2)_2_α4 LS-isoform ACh concentration-response profile produced a biphasic curve with an HS-phase potency (EC_50 (1)_ 0.51 μM) that was similar to the (α4β2)_2_β2 HS-isoform, and a LS-phase with lower ACh potency (EC_50 (2)_ 50 μM). The observed EC_50_ values are consistent with previous investigations studying α4β2 nAChR isoforms using unlinked and concatenated subunits [50, 53, 108, 109].

Expression of the α3β2 nAChR isoforms generated receptors that had very different ACh potencies (Supplemental Figure 1C). Both the α3β2 HS- and LS-isoforms produced ACh concentration-response profiles that were sigmoidal. The (α3β2)_2_β2 HS-isoform had a much higher ACh apparent potency (4.5 μM) than the (α3β2)_2_α3 LS-isoform (220 μM) (Supplemental Table 1). Expression of the (α3β4)_2_β4 HS-isoform generated nAChRs with moderate sensitivity to ACh (48 μM), while the (α3β4)_2_α3 LS-isoform produced receptors with low sensitivity to ACh (170 μM) (Supplemental Figure 1D and Supplemental Table 1).

**1.2 Expression of human α7-pHuji nAChRs in N2a cells.**

To ensure that our transfection procedure successfully transfected N2a cells with α7 nAChR plasmid DNA and resulted in plasma membrane-expressed receptors, we used the pH-sensitive probe pHuji as a reporter. 24h post pHuji-tagged α7 nAChR plasmid DNA transfection, cells were rinsed once with PBS, and imaged in a pH 7.4 pHuji-imaging buffer containing 135 mM NaCl (Sigma-Aldrich, Cat# S7653), 5 mM KCl (Sigma-Aldrich, Cat# P3911), 0.4 mM MgCl_2_ (VWR, Cat# BDH9244), 1.8 mM CaCl_2_ (Sigma-Aldrich, Cat# C8106), 20 mM HEPES (Sigma-Aldrich, Cat# H3375), and 1 mM D-glucose (Sigma-Aldrich, Cat# G8270) (67) (566 nm excitation; 598 nm emission wavelengths), using a Fluoview FV10i Laser Scanning Confocal Microscope (60X phase contrast, water-immersion, NA 1.2 objective) (Olympus, PA). The imaging regions of interest (ROIs) were saved and the pH 7.4 buffer was exchanged using a peristaltic perfusion pump for the pH 5.0 pHuji-imaging buffer (same as pH 7.4 imaging buffer, but buffered with 20 mM MES (Sigma-Aldrich, Cat# M5287) instead of HEPES) (67). After a 20min pH 5.0 buffer exchange, the same cells were imaged again. Any remaining fluorescence was from intracellular nAChRs in intracellular compartments with a neutral pH. Fluorescence values at each pH were quantified using ImageJ software (National Institutes of Health, Bethesda, MD). Cells pre- and post-quenched were hand-traced to determine their Corrected Total Cell Fluorescence (CTCF) (see Data analysis section), in the pH 7.4 and 5.0 imaging buffers to determine the extracellular and internal fluorescence of transfected cells. In addition, subtraction of post-quenched pHuji images from pre-quenched pHuji images visualized extracellular fluorescence, associated with membrane-bound α7-pHuji nAChRs (Supplemental Figure 2).

**1.3 Confocal image processing to three-dimensional projections.**

Internalization of the RVG peptide into cells was assessed through the generation of three-dimensional representations of cells labeled with the peptide. Specifically, single or small clusters of visually healthy, labeled cells were chosen for 3D visualization. These cells were imaged using microscope settings described in the *Methods*, with a slice size of 2 µm. Image stacks from each channel were initially separated and then subjected to independent deconvolution processes. Afterward, 3D projections were generated utilizing the 3D project function with interpolation. To create a comprehensive multi-channel 3D model, the phase and fluorescent channels were merged into a single image (Supplemental Figure 3).

# Supplementary Figures and Tables

## Supplementary Table

**Supplemental Table 1. Fitted parameters of the α7, α4β2α5, α6/α3β2β3, β3-α6-β2-α4-β2, (α4β2)_2_β2, (α4β2)_2_α4, (α3β2)_2_β2, (α3β2)_2_α3, (α3β4)_2_β4 and (α3β4)_2_α3 nAChR ACh concentration-response profiles.** The ng of injected cRNAs for each transcript that resulted in robust nAChR expression are shown. ACh concentration-response curves were fit using nonlinear regression analysis, and determined parameters are shown with 95% confidence intervals.

| **nAChR Subtype** | **ng of cRNA Injected per Transcript** | **N (n)** | **EC_50 (1)_ (μM)**  **(95% CI)** | **N_H (1)_**  **(95% CI)** | **EC_50 (2)_ (μM)**  **(95% CI)** | **N_H (2)_ (95% CI)** |
| --- | --- | --- | --- | --- | --- | --- |
| α7 | 40 | 6 (11) | 140  (130 – 160) | 1.6  (1.4 – 1.9) | N/A | N/A |
| α4β2α5 | 2.5 : 2.5 : 25  α4 : β2 : α5 | 4 (10) | 1.1  (0.92 – 1.2) | 0.90  (0.82 – 0.99) | N/A | N/A |
| α6/α3β2β3 | 12 : 12 : 6  α6/α3 : β2 : β3 | 4 (8) | 4.5  (3.4 – 5.7) | 0.74  (0.62 – 0.86) | N/A | N/A |
| β3-α6-β2-α4-β2 | 30 | 4 (6) | 1.5  (1.3 – 1.8) | 0.77  (0.69 – 0.85) | N/A | N/A |
| (α4β2)_2_β2 | 1 : 30  α4 : β2 | 3 (6) | 3.9  (3.3 – 4.5) | 0.85  (0.76 – 0.95) | N/A | N/A |
| (α4β2)_2_α4 | 12.5 : 0.125  α4 : β2 | 3 (3) | 0.51  (0.18 – 1.4) | = 1 | 50  (35 – 78) | =1 |
| (α3β2)_2_β2 | 1 : 30  α3 : β2 | 6 (18) | 4.5  (3.7 – 5.2) | 0.95  (0.84 – 1.1) | N/A | N/A |
| (α3β2)_2_α3 | 30 : 1  α3 : β2 | 5 (16) | 220  (180 – 270) | 0.70  (0.63 – 0.77) | N/A | N/A |
| (α3β4)_2_β4 | 1 : 30  α3 : β4 | 4 (12) | 48  (41 – 54) | 1.0  (0.92 – 1.2) | N/A | N/A |
| (α3β4)_2_α3 | 30 : 1  α3 : β4 | 6 (12) | 170  (160 – 180) | 1.9  (1.7 – 2.1) | N/A | N/A |

## Supplementary Figure

**
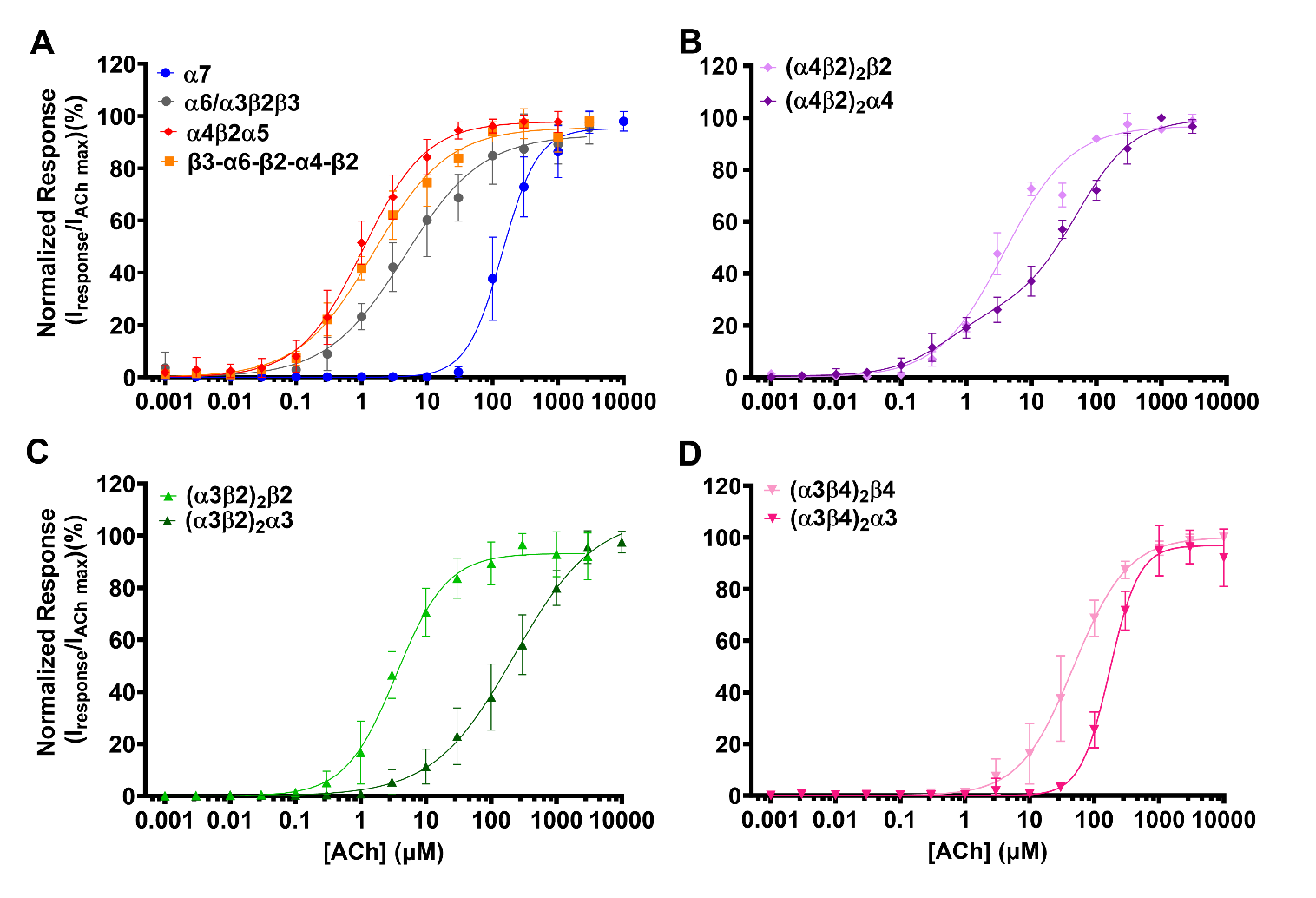
**

**Supplemental Figure 1. ACh concentration-response profiles demonstrate the expression of nAChR subtypes and isoforms.** *Xenopus laevis* oocytes injected with unbiased or biased single nAChR subunit ratios, or concatenated (β3-α6-β2-α4-β2) cRNAs were exposed to 1s applications of increasing concentrations of ACh. **A)** ACh response profiles for nAChR subtypes lacking isoforms. **B)** The (α4β2)_2_β2 ACh concentration-response curve was best fit with a monophasic sigmoidal dose-response curve, while the (α4β2)_2_α4 isoform was best fit with a biphasic profile. **C)** The α3β2 and **D)** α3β4 nAChR subtypes ACh concentration-response profiles demonstrating expression of HS- and LS-isoforms. EC_50_ values and n_H_ values are reported in Supplemental Table 1. Points are the mean ± S.D. (N =3 - 6, n = 3 - 18).


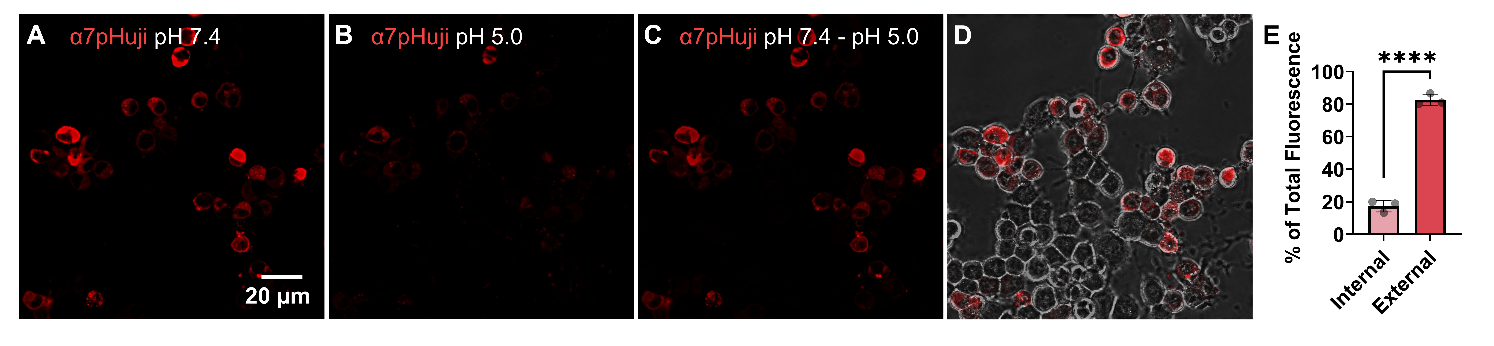


**Supplemental Figure 2. α7-pHuji nAChR transfection of N2a cells confirms localization of receptors at the cell plasma membrane.** N2a cells were transfected with α7-pHuji nAChR plasmid DNA and live-cell imaged to observe the location of the tagged receptors. **A)** N2a cells transfected with α7-pHuji plasmid DNA imaged in pH 7.4 buffer. **B)** The same N2a cells imaged in pH 5.0 buffer, showing only receptors expressed intracellularly. **C)** Subtraction image of A) and B), representing α7-pHuji receptors expressed on the plasma membrane. **D)** Same image as C with phase channel. **E)** CTCF analysis of α7-pHuji nAChR transfected N2a cells at pH 7.4 and pH 5.0, showing significant amounts of α7-pHuji nAChRs are localized to the plasma membrane of N2a cells. Data are mean ± S.D. (N = 3, n = 90, ****p < 0.0001, unpaired two-tailed t-test).


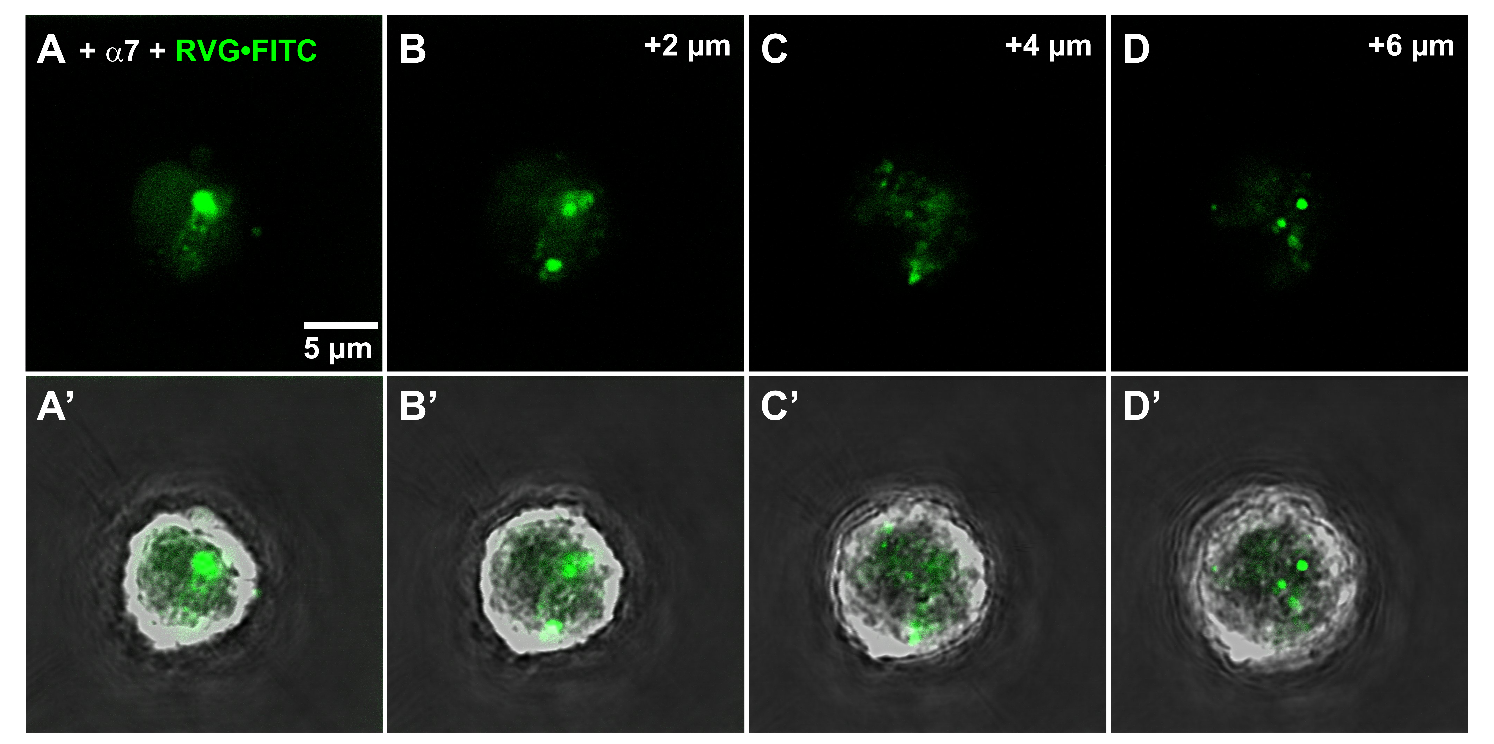


**Supplemental Figure 3. RVG-FITC peptide internalizes into cells expressing α7 nAChRs. A)** Representative 3D projection from z-stack confocal images of α7 nAChR-transfected N2a cells treated with 30 μM FITC-tagged RVG peptide (z-stack slice size: 2 μm). **B - D)** Same as A), each representing another z-stack slice with a 2 μm distance from the previous image, moving through the cell. **A’ - D’)** Same as A - D with the phase channel. RVG-FITC shows localization to the intracellular space in α7 nAChR-transfected N2a cells.
